# Comparative genomics of *Aspergillus nidulans* and section *Nidulantes*

**DOI:** 10.1016/j.crmicr.2025.100342

**Published:** 2025-01-16

**Authors:** Sebastian Theobald, Tammi Vesth, Jane L. Nybo, Jens C. Frisvad, Inge Kjærbølling, Stephen Mondo, Kurt LaButti, Sajeet Haridas, Robert Riley, Alan A. Kuo, Asaf A. Salamov, Jasmyn Pangilinan, Anna Lipzen, Maxim Koriabine, Mi Yan, Kerrie Barry, Alicia Clum, Ellen K. Lyhne, Elodie Drula, Ad Wiebenga, Astrid Müller, Ronnie J.M. Lubbers, Roland S. Kun, Ana Carolina dos Santos Gomes, Miia R. Mäkelä, Bernard Henrissat, Blake A. Simmons, Jon K. Magnuson, Jakob B. Hoof, Uffe H. Mortensen, Paul S. Dyer, Michelle Momany, Thomas O. Larsen, Igor V Grigoriev, Scott E. Baker, Ronald P. de Vries, Mikael R. Andersen

**Affiliations:** a Department of Biotechnology and Biomedicine, Technical University of Denmark, DK-2800 Kongens Lyngby, Denmark; b Department of Energy Joint Genome Institute, Lawrence Berkeley National Laboratory, Berkeley, CA 94720, USA; c Architecture et Fonction des Macromolécules Biologiques (AFMB), Marseille, France; d Biodiversité et Biotechnologie Fongiques, UMR 1163, INRAE, Marseille, France; e Fungal Physiology, Westerdijk Fungal Biodiversity Institute, Utrecht, The Netherlands; f Department of Microbiology, University of Helsinki, Finland; g US Department of Energy Joint Bioenergy Institute, Berkeley, CA, USA; h School of Life Sciences, University of Nottingham, Nottingham NG7 2RD, UK; i Fungal Biology Group & Plant Biology Department, University of Georgia, Athens, Georgia, USA 30602; j Department of Plant and Microbial Biology, University of California Berkeley, Berkeley, CA 94720, USA; k Earth and Biological Sciences Directorate, Pacific Northwest National Laboratory, Richland, USA

**Keywords:** *Aspergillus*, Comparative genomics, *Nidulantes*, Secondary metabolism, CAZy

## Abstract

*Aspergillus nidulans* is an important model organism for eukaryotic biology and the reference for the section *Nidulantes* in comparative studies. In this study, we *de novo* sequenced the genomes of 25 species of this section. Whole-genome phylogeny of 34 *Aspergillus* species and *Penicillium chrysogenum* clarifies the position of clades inside section *Nidulantes*. Comparative genomics reveals a high genetic diversity between species with 684 up to 2433 unique protein families. Furthermore, we categorized 2118 secondary metabolite gene clusters (SMGC) into 603 families across Aspergilli, with at least 40 % of the families shared between *Nidulantes* species. Genetic dereplication of SMGC and subsequent synteny analysis provides evidence for horizontal gene transfer of a SMGC. Proteins that have been investigated in *A. nidulans* as well as its SMGC families are generally present in the section *Nidulantes*, supporting its role as model organism. The set of genes encoding plant biomass-related CAZymes is highly conserved in section *Nidulantes*, while there is remarkable diversity of organization of MAT-*loci* both within and between the different clades. This study provides a deeper understanding of the genomic conservation and diversity of this section and supports the position of *A. nidulans* as a reference species for cell biology.

## Highlights

• *Aspergillus* section *Nidulantes* demonstrates high genomic diversity.
• At least 40 % of the secondary metabolite clusters are shared within the section.
• The CAZyme gene content is highly conserved in section *Nidulantes.*
• Section *Nidulantes* has a diverse organization of the mating type loci.
• *A. nidulans* was confirmed to be a good reference species for the section.

## Graphical abstract

**Figure:**
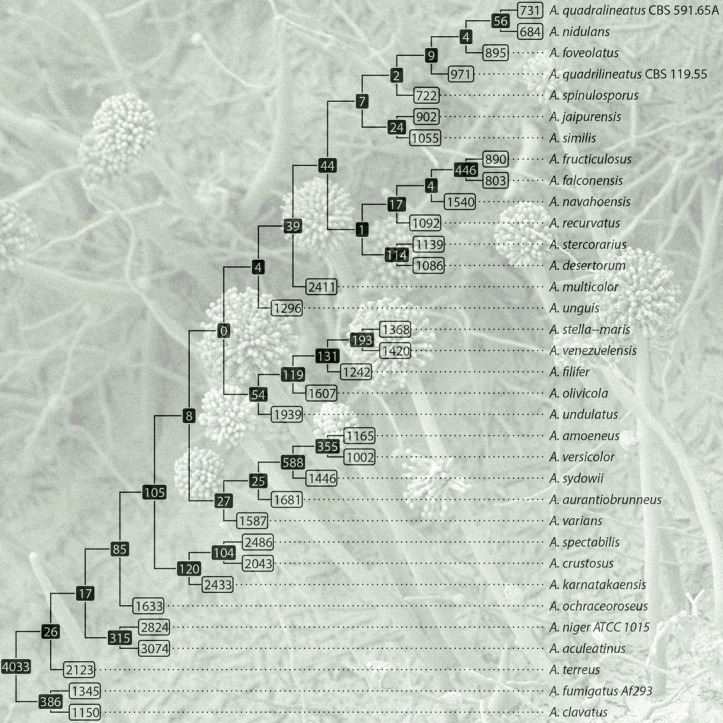

## 1 Introduction

Research on *Aspergillus nidulans* has benefited the general scientific community in many ways. Its use as a model organism for eukaryotic biology has provided important insights into cell polarity and cell cycle mechanisms (Momany, 2002), DNA repair mechanisms (Goldman and Kafer, 2004), morphogenesis (Virag et al., 2007; Ojeda-López et al., 2018) and cytoskeleton development (Shukla et al., 2017). In addition, studies on *A. nidulans* have added important information to the understanding of anti-fungal drug resistance (de Waard and van Nistelrooy, 1979; Osherov et al., 2001; Rocha et al., 2002). *A. nidulans* is part of section *Nidulantes* which has been synonymized with section *Versicolores* and *Aenei* — defining them as subclades (officially series) of section *Nidulantes* (Hubka et al., 2016). Members of the section are mainly decomposers of plant material, but also include species associated with human infections (Baddley et al., 2009) in the case of *A. nidulans* (immunocompromised patients only) *A. unguis* and *A. versicolor* — the latter affecting indoor environments (Engelhart et al., 2002) while also being used as a source for xylanases (Carmona et al., 2005). The section also includes the coral pathogen *A. sydowii* (Alker et al., 2001).

Besides their morphological characteristics, the secondary metabolites (SMs) they produce are characteristic to species of this section. Sterigmatocystin, a carcinogen (Terao, 1983) and common food contaminant (Veršilovskis and De Saeger, 2010), can be produced throughout many subgenera, but is mostly present in aspergilli of section *Nidulantes* and *Circumdati* (Rank et al., 2011). Besides mycotoxins, some species also produce medically relevant SMs, such as penicillin, albeit not commercially (Raper, 2006; Holt and MacDonald, 1968; Díez et al., 1990). The importance of SMs has stimulated much interest in the investigation of secondary metabolite gene clusters (SMGCs) (Nielsen et al., 2011; Klejnstrup et al., 2012; Sanchez et al., 2010).

Despite the extensive use of *A. nidulans* as a fungal model organism, its suitability as an appropriate model for *Aspergillus* and filamentous fungi in general remains to be established. Genome sequencing of the *A. nidulans* genome and comparisons to *A. fumigatus* and *A. oryzae* (Galagan et al., 2005) have highlighted similarities between these fungi but did not encompass sufficient species to prove the general applicability of *A. nidulans* as a model. In this study, we investigate the dynamics of selected homologous protein families of 34 *Aspergillus* (and *P. chrysogenum*) species to assess the application of *A. nidulans* as a model organism for these species. We provide further insights into the phylogeny of section *Nidulantes* using whole-genome-phylogenetic approaches and investigate in particular the diversity of secondary metabolite gene clusters (SMGCs), conservation of genes encoding plant biomass-related CAZymes, and dynamics of mating-type (*MAT*) *locus* organization.

## 2 Materials and methods

### 2.1 Purification of DNA

For all genome sequences generated in this study, conidia stored at -80 °C were used to inoculate solid Czapek Yeast Extract agar (CYA) medium. Fresh conidia were harvested after 7–10 days and suspended in a 0.1 % Tween solution. For generation of biomass, liquid CYA medium was inoculated and cultivated for 5–10 days at 30 °C. Mycelium was isolated by filtering through Miracloth (Millipore, 475855-1R), freeze dried and stored at -80 °C until further use. DNA isolation was performed using a modified version of standard phenol extraction (Kjærbølling et al., 2018) and checked for quality and concentration using a NanoDrop (BioNordika, DK).

### 2.2 DNA sequencing and assembly

All genomes in this study were sequenced with Illumina technologies. For all genomic Illumina libraries, 100 ng of DNA was sheared to 270 bp fragments using the Covaris LE220 (Covaris) and size selected using SPRI beads (Beckman Coulter). The fragments were treated with end-repair, A- tailing, and ligated to Illumina compatible adapters (IDT, Inc) using the KAPA-Illumina library creation kit (KAPA biosystems). The prepared libraries were quantified using KAPA Biosystem's next-generation sequencing library qPCR kit and run on a Roche LightCycler 480 real-time PCR instrument. The quantified libraries were then multiplexed with other libraries, and library pools were prepared for sequencing on the Illumina HiSeq sequencing platform utilizing a TruSeq paired-end cluster kit, v3, and Illumina's cBot instrument to generate clustered flow cells for sequencing. Sequencing of the flow cells was performed on the Illumina HiSeq2000 sequencer using a TruSeq SBS sequencing kit, v3, following a 2 × 150 indexed run recipe. After sequencing, the genomic fastq files were QC filtered to remove artefacts/process contamination and assembled using Velvet (Zerbino and Birney, 2008). The resulting assemblies were used to create *in silico* long mate-pair libraries with inserts of 3000 ± 90 bp that were then assembled with the target fastq using AllPathsLG release version R47710 (Gnerre et al., 2011). All genomes were annotated using the JGI annotation Pipeline and are available at the JGI fungal genome portal MycoCosm (Grigoriev et al., 2014) (http://mycocosm.jgi.doe.gov/fungi).

### 2.3 Data collection

Predicted genes, protein sequences and annotations (e.g., from SMURF, InterPro, and Gene Ontology) for additional genomes compared with the genomes from this study were collected from JGI (https://mycocosm.jgi.doe.gov/). A customized version of SMURF (Khaldi et al., 2010) was used to annotate secondary metabolite gene clusters throughout draft *Aspergillus* genomes.

### 2.4 Creating protein families

Protein families were created using single linkage on bidirectional protein BLAST (Camacho et al., 2009) hits with a percent identity of at least 50 % and sum of coverage (query and subject) of at least 130 % (Vesth et al., 2018).

### 2.5 Whole genome phylogeny

Whole genome phylogeny was constructed from alignment of 200 bidirectional best hits between species using RAxML (Stamatakis, 2014), MAFFT (Katoh et al., 2002), and Gblocks Castresana, 2000).

### 2.6 Prediction of encoded CAZymes

CAZymes were predicted for all genomes using the Carbohydrate-Active Enzymes database routines (www.cazy.org, (Drula et al., 2022)) and methods described in (Vesth et al., 2018).

### 2.7 Creating SMGC families

SMGC families were created according to (Vesth et al., 2018). In brief, bidirectional blast hits between proteins of secondary metabolite gene clusters were aggregated into a cluster vs cluster similarity score network. Subsequently, random walk clustering was used on this network to create SMGC families (Vesth et al., 2018).

### 2.8 Genetic dereplication

Data from the the Minimum Information on Biosynthetic Gene clusters (MIBiG) database (Medema et al., 2015) was extracted using biopython (Cock et al., 2009) and subset for entries from *Aspergillus* and *Penicillium* species. Selected entries were compared to secondary metabolite proteins of the dataset using protein BLAST (Camacho et al., 2009) and best hits, which pass a cutoff of 95 % protein sequence identity, 95 % query coverage and 95 % subject coverage cutoff.

### 2.9 ML phylogenies of NRPS

Protein sequences were aligned using clustalo (Sievers et al., 2011), trimmed with trimal (Capella-Gutiérrez et al., 2009) using a gap threshold of 0.8, a similarity threshold of 0.001, keeping 80 % of columns. ML phylogenies were created using iqtree (Nguyen et al., 2015) with substitution model chosen according to ModelFinder Plus (Kalyaanamoorthy et al., 2017) and 1000 times ultrafast bootstrap (Minh et al., 2013).

## 3 Results and discussion

### 3.1 Genome statistics of section *Nidulantes* represent a range similar to that of the whole genus *Aspergillus*

**Fig. 1:**
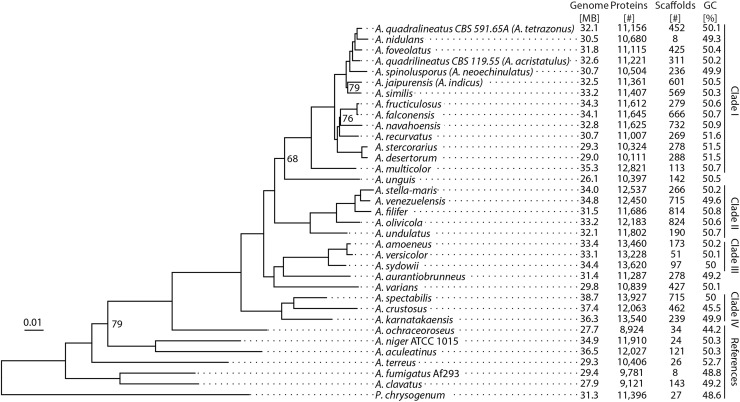
Dendrogram of species. Whole genome phylogeny constructed from alignment of 200 bidirectional best hits between species using RAxML (Stamatakis, 2014), MAFFT (Katoh et al., 2002), and Gblocks (Castresana, 2000). Bootstrap support shown at node if under 80. Genome statistics is shown in right panel.

As a first step, we examined the overall variation of genome size and content to see how the 25 new genomes of section *Nidulantes* compared to the rest of genus *Aspergillus*. Genome sizes in the species of *Nidulantes* range from 26.1 to 38.7 Mb, with a GC content of 45.5–50.8 % (Fig. 1), which is similar to other aspergilli as shown here and in previous studies (de Vries et al., 2017), although *A. unguis* has an uncharacteristically small genome (26.1 Mb). Even so, the gene density of *A. unguis* is very high, as the number of predicted genes is 10,397, which is well within the typical range.

In summary, *A. nidulans* is typical of the section *Nidulantes*, but overall section *Nidulantes* shows substantial diversity.

### 3.2 A 200-gene phylogeny shows four distinct clades in section *Nidulantes*

Next, we wanted to establish a clear phylogeny for section *Nidulantes*, as previous studies have disagreed on the boundaries of the section (Hubka et al., 2016; Chen et al., 2016). Resolution of the evolutionary history of the section is of importance for studies of gene and genome diversity and classical taxonomy. We sought to improve the section definitions and species relationships using whole-genome phylogenetic methods.

As such, we used 200 concatenated genes to construct a maximum likelihood (ML) phylogram of 28 members of section *Nidulantes* and seven references species outside the section (Fig. 1). Nearly all nodes were resolved with bootstrap values above 80 %.

Examining the phylogram, section *Nidulantes* divides into four major clades with three species (*A. unguis, A. aurantiobrunneus*, and *A. varians*) outside of these clades. Previous studies (Hubka et al., 2016; Chen et al., 2016) have generated similar results from single-gene phylograms based on coding regions of the genes for beta-tubulin (*benA*), calmodulin (*caM*), RNA polymerase II (*RPB2*), and internal transcribed spacer (ITS) sequences. The present study detected similar clades, although with some slight differences. *Aspergillus nidulans* is found in Clade I, but two isolates of *A. quadrilineatus*, which were previously seen as separate species, span the clade, and show more variation than between other species. This does not support the previous suggestion to combine *A. tetrazonus* and *A. acristulatus* into *A. quadrilineatus*. Interestingly the phylogram shows that members of Clade IV (Fig. 1), which was previously described as section *Aenei*, have larger genomes than other *Nidulantes* and *Aspergillus* species (37.5 Mb on average). Clade IV is also found to locate outside of the rest of section *Nidulantes*, in accordance with previous work (Chen et al., 2016).

### 3.3 The section *Nidulantes* pan- and core genome shows that even closely related species have a large unique genome content

**Fig. 2:**
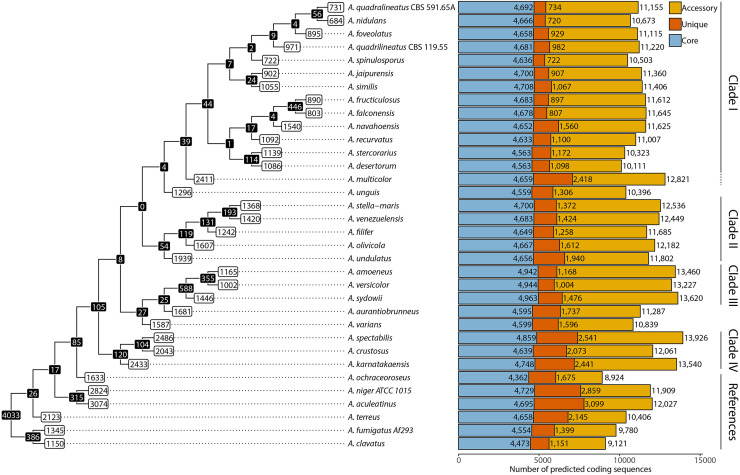
Core, accessory, and unique proteins found throughout *Aspergillus* species. Protein families were built using single linkage on bidirectional protein blast hits with a percent identity of at least 50 % and sum of coverage (query and subject) of at least 130 %. Left: Numbers on nodes show counts of unique protein families for species included in the branch; Numbers on tips show counts of unique protein families for individual organisms. Right: Core, unique and accessory proteins represented in stacked bars per genome.

In order to quantify and identify the diversity of the species of section *Nidulantes* at the genetic level, as well as further evaluate how similar *A. nidulans* is to the other species, we decided to determine the pan-, core-, accessory, and unique genome content of the 25 new genomes as well as reference species. We did this by determining families of homologous proteins (a "protein family") across 34 species in total (Fig. 2). A protein family is seen as a set of homologous predicted protein sequences across genomes. These families were then compared to the phylogenetic tree as determined above (Fig. 1).

In total, 4,033 protein families were found to be present in all 34 *Aspergillus* genomes included in the present study (the *Aspergillus* core proteome). As these protein families could have more than one member per species (i.e., hypothetical paralogs), the number of proteins in the core proteome ranges from 4,362 to 4,963 proteins (Fig. 2).

Regarding section *Nidulantes*-specific content, 105 protein families were found exclusively throughout all members of the section that were not found in other aspergilli, which is rather low compared to a study of similar size for section *Nigri*, where 250 protein families were shared exclusively by members of the Section (Vesth et al., 2018). By contrast, the section *Nidulantes* has a relatively large number of genes found only in specific genomes (ranging from 684-2486 per species) (Fig. 2, white boxes) although not a large number of genes shared within clades. Particularly in the relatively closely related species in Clade I, the numbers are low (Fig. 2, black boxes).

### 3.4 Pan- and core genome analysis confirms phylogenetic clades and suggests species identity of *A. fructiculosus* and *A. falconensis*

Overall, the pan, auxiliary, and core genome analysis support the presence of four major clades and three species not found in the clades (Fig. 1). However, the presence of 2411 species-unique protein families in *A. multicolor* was surprisingly high within Clade 1 and this fact, combined with its localization in the phylogenetic tree with *A. unguis*, suggests that it could be placed outside clade I.

A further point of attention in Clade I is the large number (446) of protein families unique to *A. fructiculosus* and *A. falconensis*. In combination with the whole genome phylogeny (Fig. 1), this high number suggests that they could be different isolates of the same species. However, a similar analysis for *A. niger* isolates (Vesth et al., 2018) which were sequenced and annotated with the same method, showed a lower number of unique protein families (182-424) for isolates from the same species, whereas here both isolates have more than 800 unique protein families. However, an even higher average number of 1163 unique genes has been described for *A. fumigatus* (Lofgren et al., 2022).

### 3.5 *A. nidulans* is genetically representative of Clade I of section *Nidulantes*

Examining whether the eukaryotic model organism *A. nidulans* is a typical representative of section *Nidulantes* in general, it is clear from the analysis above that there is quite a large genetic diversity in the section. However, as can be seen from the shared protein families in Fig. 2 and the phylogenetic distances of Fig. 1, *A. nidulans* is quite closely related to the members of Clade I, with the closest relative being *A. quadrilineatus* CBS 591.65A. Furthermore, *A. nidulans* only contains 684 protein families which are species-unique, suggesting that much of its proteome is representative of section *Nidulantes*.

### 3.6 Well-known regulatory proteins from *A. nidulans* show variation in conservation across section *Nidulantes*

**Fig. 3:**
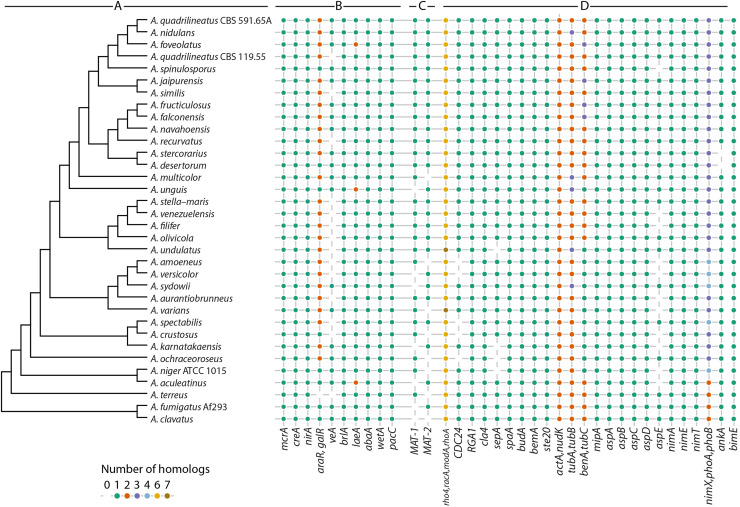
**A.** Cladogram of section *Nidulantes* and six reference aspergilli. **B.** Selection of protein families with known representatives in the dataset. Column labels correspond to annotated *A. nidulans* genes. **C.** Orthologs of mating factors MAT-1 and MAT-2. **D.** Distribution of polarity, cytoskeleton and cell cycle proteins throughout the dataset. Protein families for *A. nidulans* proteins (one family per column) involved in polarity, cytoskeleton and cell cycle were extracted from protein families and annotations concatenated if multiple annotated proteins were found per family (annotated on the x-axis). Homologs are shown for all species of the dataset. Number of homologs is shown by point color.

As *A. nidulans* is a model system for fungal biology, we wanted to evaluate whether well-known regulatory proteins studied in *A. nidulans* are found in other members of section *Nidulantes* and in reference species. For this, we selected the carbon catabolite repressor CreA (de Vries et al., 2017; Shroff et al., 1996), nitrogen regulator NirA (Rand and Arst, 1978), pH response regulator PacC (Denison, 2000), master regulators of secondary metabolism McrA and LaeA (Bok and Keller, 2004; Oakley et al., 2017), and conidiophore development regulator BrlA (Adams et al., 1988) and other proteins, as well as mating factors MAT-1 and MAT-2 (Paoletti et al., 2007). Using the protein families described above, we investigated whether these regulators are part of the core or accessory proteome (Fig. 3B).

Overall, most metabolic regulators (CreA, PacC, McrA) and morphology regulators (WetA, AbaA, BrlA) are conserved across both section *Nidulantes* and genus *Aspergillus* with a single ortholog per genome (Fig. 3B). However, a few stand out.

VeA, a factor involved in the response to light in *A. nidulans* (Bayram and Braus, 2012; Mooney et al., 1990), is not found in 13 species of section *Nidulantes* using a synteny-based analysis. The analysis also shows it to be absent in *A. terreus* based on the criteria used in the present study (i.e., at least 50 % and sum of coverage at least 130 % (Vesth et al., 2018)), but a syntenic ortholog with only 37 % sequence identity can be found in that genome.

LaeA interestingly has duplications in three species: *A. unguis, A. foveolatus,* and *A. aculeatinus*. We examined the duplication using a maximum likelihood-based phylogram (Fig. S1), which shows mixed origins of the extra copies: The second copy in *A. foveolatus* (Clade I) is more closely related to orthologs from clade II. *A. aculeatinus* seems to contain a *laeA* homolog more closely related to that of *A. terreus*, and the second copy in *A. unguis* only has a close homolog in *A. croceus*.

Finally, L-arabinose and D-galactose-utilization regulators (AraR and GalR) could not be resolved into separate families. This concurs with the original discovery of GalR as an uncharacterized paralog of AraR in *A. nidulans* (Christensen et al., 2011), with little conservation in other ascomycetes. A phylogram of this family (Fig. S2), shows that GalR has orthologs in all members of section *Nidulantes* except *A. unguis* (Fig. 3B). Also, sequence variation in GalR is much higher than in AraR, which makes it difficult to see, whether the extra homolog of AraR in *A. ochraceoroseus* is more likely to be GalR or even a third function.

### 3.7 Examination of proteins involved in mating and sexual development reveals unexpected variation in section *Nidulantes* and the genus *Aspergillus*

**Fig. 4:**
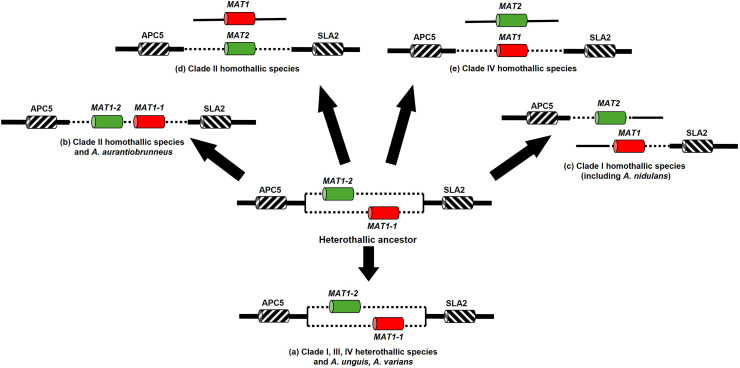
Organization of MAT *loci* in the section *Nidulantes*. Location shown (not to scale) of mating-type genes MAT-1 (alpha domain; in red) and/or MAT-2 (HMG domain; in green) relative to flanking apc5 (anaphase promoting complex) and sla2 (cytoskeleton assembly control) genes. Dotted lines indicate idiomorph region whilst bold lines indicate conserved flanking sequence. Five different arrangements of MAT *loci* are thought to have evolved from an ancient heterothallic ancestor and are now present in Clade I-IV species as indicated. Partially adapted from (Ojeda-López et al., 2018).

*A. nidulans* has been a model for sexual development in filamentous ascomycetes (Dyer and O'Gorman, 2012) and as such, we wanted to see how the conservation of mating factors and proteins involved in sexual development varied across the section *Nidulantes* and genus *Aspergillus*. Our analysis revealed an unexpectedly high diversity of five different forms of organization of MAT-*loci* both within and between the different clades of the section *Nidulantes* (Fig. 3C; Fig. 4). It is thought that the ancestral sexual reproductive mode within the aspergilli as a whole is heterothallism (obligate outbreeding), with a single mating-type (MAT) *locus* present (with two different idiomorphs MAT1-1 and MAT1-2) neighbored by conserved genes that can include *sla2, apn2*, and *apc5* which show synteny across species (Ojeda-López et al., 2018). In the present study a typical MAT *locus* containing either a *MAT1-1* or *MAT1-2* gene bordered upstream and downstream by *apc5* and *sla2*, respectively, was detected for eight species of the section *Nidulantes*, consistent with heterothallism as already reported for *A. heterothallicus* from this section (Ojeda-López et al., 2018). This included all of the more basal Clade III and most Clade IV species (Fig. 1), suggesting a heterothallic ancestor for the section. By contrast the remaining 20 species contained both *MAT-1* and *MAT-2* genes in the genome, consistent with homothallism (self-fertility) (Ojeda-López et al., 2018, Paoletti et al., 2007)) as reported so far for many but not all of these species (Raper et al., 1965). In the case of *A. nidulans*, the two mating-type genes are present in different regions of the genome as previously reported (Galagan et al., 2005; Paoletti et al., 2007), with *MAT-1* linked to *sla2* whereas *MAT-2* was linked with *apc5* (Fig. 4). The same arrangement of *MAT loci* was seen in all other Clade I isolates, except for the most basal *A. multicolor* which appeared heterothallic (Fig. 1). However, a different organization of homothallic MAT *loci* was seen elsewhere in the section. Clade II species either contained *MAT-1* and *MAT-2* co-located at the same single MAT *locus* bordered by *apc5* and *sla2*, or contained only *MAT-2* bordered by *apc5* and *sla2*, with *MAT-1* located elsewhere in the genome (Fig. 4). Meanwhile, for one putative homothallic Clade IV species, *A. spectabilis, MAT-1* was bordered by *apc5* and *sla2*, whilst it was instead *MAT-2* that was located elsewhere in the genome. Thus, MAT *loci* in the section *Nidulantes* appeared to be remarkably dynamic region(s) with different arrangements of *MAT-1* and *MAT-2*, and associated reproductive modes, perhaps selected for by evolution according to different ecological niches (Ojeda-López et al., 2018).

A further analysis was then made of orthologs for an additional 84 proteins involved in mating and sexual development including those involved in detection of environmental signals, pheromone signaling, transcriptional activation, and cleistothecia and ascospore development (Dyer and O'Gorman, 2012). This revealed that for the most part gene content was highly conserved across all members of the section, consistent with *A. nidulans* being a suitable model for studies of sexual development (Fig. S3). However, there were some intriguing deviations. As previously mentioned, a *veA* gene homolog appeared to be absent from 13 of the study species, which was surprising given that VeA has an important regulatory role in sexual development in the aspergilli and beyond and that in contrast VelB and VelC homologs were uniformly present (Bayram and Braus, 2012; Dyer and O'Gorman, 2012). There were also variable number of *ppo* oxylipin biosynthesis genes present, with between 2-4 genes detected per species, together with variation in content of recently identified high mobility group (HMG) sexual transcription factors (N Salih and P Dyer, unpublished data). A *ppgA* pheromone precursor gene was also apparently absent from 3 species in the section, again unexpected given the supposed requirement for pheromone signaling during sexual reproduction in the aspergilli (Paolette et al., 2007; Seo et al., 2004). It is noted that a *ppgB* homolog has very recently been reported (Krappmann et al., 2025) and it will be interesting to study the distribution in *Aspergillus* genomes in future studies.

One other unusual feature of the section *Nidulantes* not seen directly in other aspergilli is the formation of specialized Hülle cells, which surround developing cleistothecia and are thought to act as nursing cells (Dyer and O'Gorman, 2012; Liu et al., 2021). The genetic basis of Hülle formation remains unknown but it is speculated that some of the 105 section-specific protein families might be involved in this process.

### 3.8 *A. nidulans* is a representative model for studies of polarity, cytoskeleton and cell cycle

The highly polar growth of filamentous fungi makes them ideal for studies of morphology and its coordination with the cell cycle. The spatial separation of nuclei and well-developed genetic system of *A. nidulans* has made it an attractive model for many of these studies (Momany, 2002; Harris and Momany, 2004; Riquelme, 2013).

To investigate whether *A. nidulans* is a good model for morphology and cell cycle in other aspergilli, we identified homologs of 28 key polarity proteins including the Rho GTPase Cdc42 (ModA), its associated guanine nucleotide exchange factor (GEF) Cdc24 and GTPase-activating protein (GAP) Rga1, and the p21-activated kinase Cla4. We also identified homologs of cytoskeletal genes including actin, tubulins and septins and key cell cycle genes including cyclin dependent kinases, wee1 kinase, phosphatases and anaphase promoting complex (APC) components (Fig. 3D).

As expected, multiple Cdc42 homologs were present in all aspergilli examined and included Cdc42, RacA, Rho4, and RhoA along with 2-3 other uncharacterized Rho GTPases. Single homologs of Rga1 and Cla4 were also present in all species examined. While most of the species examined had a single Cdc24 GEF homologue, five species of clade III and IV lacked a Cdc24 GEF. This is surprising because Cdc24 is an essential gene in *A. nidulans* (Shuman and Momany, 2022; Si et al., 2016). The lack of Cdc24 homologs in five of the 34 species examined, along with conservation of other key polarity genes, suggests that a more diverged gene might encode a GEF for Cdc42 in these species.

Single homologs of polarisome components SpaA, BudA, BemA, and Ste20 were present in all species, while four lacked the formin SepA (Harris and Moimany, 2004) in their annotation. All species contained single or multiple homologs of cytoskeletal elements actin, actin related protein Arp1 (*nudK*), and tubulins. Alpha and beta tubulins ranged from 1-3 homologs per species, consistent with previously reported presence of tubulins specialized for specific developmental states (Doshi et al., 1991; Kirk and Morris, 1991). The core septins AspA^CDC11^, AspB^CDC3^, AspC^CDC12^, and AspD^CDC10^ had single homologs in each species, consistent with the assembly of these core septins into complexes (Pan et al., 2007). Strikingly, the noncore septin AspE was absent in 11 of the 34 species examined. This is similar to the patchy distribution of this nonessential septin across fungi and across kingdoms (Shuman and Momany, 2022; Pan et al., 2007).

All of the key cell cycle genes examined had at least one homolog present in all species with the exception of the wee1 kinase AnkA which was absent in two clade I species. AnkA is essential in *A. nidulans* (Pan et al., 2007), so its absence in some species is unexpected and once again suggests that a gene that is too diverged to be detected might play the same role in these species.

In terms of general trends, we found that extra homologs of polarity, cytoskeletal and cell cycle genes were common across the *Aspergillus* species examined, with all 34 species examined showing the gain of 1–3 homologs. Complete loss of all homologs of a specific gene was less common, occurring in 16/34 species examined, with the nonessential septin AspE accounting for half of the cases. Loss of a gene family was most common in clades III and IV. *A. nidulans* contained representatives of each polarity, cytoskeletal, or cell cycle gene examined and so in terms of gene content is a good model for all the *Aspergillus* species examined.

### 3.9 The CAZyme gene content of the genomes of section *Nidulantes* shows a high level of conservation, especially between closely related species

The ability to efficiently degrade a broad range of plant biomass substrates is one of the main characteristics that drew attention to species of the genus *Aspergillus* (Culleton et al., 2013). However, section *Nidulantes* is not often studied in this context, unlike e.g., section *Nigri* (Vesth et al., 2018). Studies on plant biomass conversion in section *Nidulantes* largely center around the best studied species, *A. nidulans*. Many CAZymes have been purified from this species as well as their corresponding genes cloned (Culleton et al., 2013; de Vries and Visser, 2001), and studies into the regulation of production of these enzymes have been performed (Coradetti et al., 2012; Klaubauf et al., 2014; Pardo and Orejas, 2014). In addition, (post-)genomic studies have delved deeper into the process of plant biomass degradation by *A. nidulans*, such as a time-course exoproteome analysis (Sayhedkar et al., 2012) and a comparison of the secretome during growth on different starch substrates (Neliunaite et al., 2016). In comparison, only a small number of studies addressed this topic in other species of section *Nidulantes*, such as *A. versicolor* (Carmona et al., 2005; Arana-Cuenca et al., 2019; Jeya et al., 2005; Lee et al., 2011; de Vargas Andrade et al., 2004; Somera et al., 2009; Aragon et al., 2013), *A. quadrilineatus* (Suryawanshi et al., 2019), and *A. unguis* (Shruthi et al., 2019; Rajasree et al., 2013).

To obtain a better understanding of the plant biomass degrading potential across section *Nidulantes*, a comparative analysis of the CAZyme genes related to plant biomass degradation in the genomes of the species within section *Nidulantes* and a set of control species was performed. This analysis was also compared to the growth profiles of the species on 33 plant biomass related mono-, di- and polysaccharides, lignin, and crude substrates (Fig. S4).

**Fig. 5:**
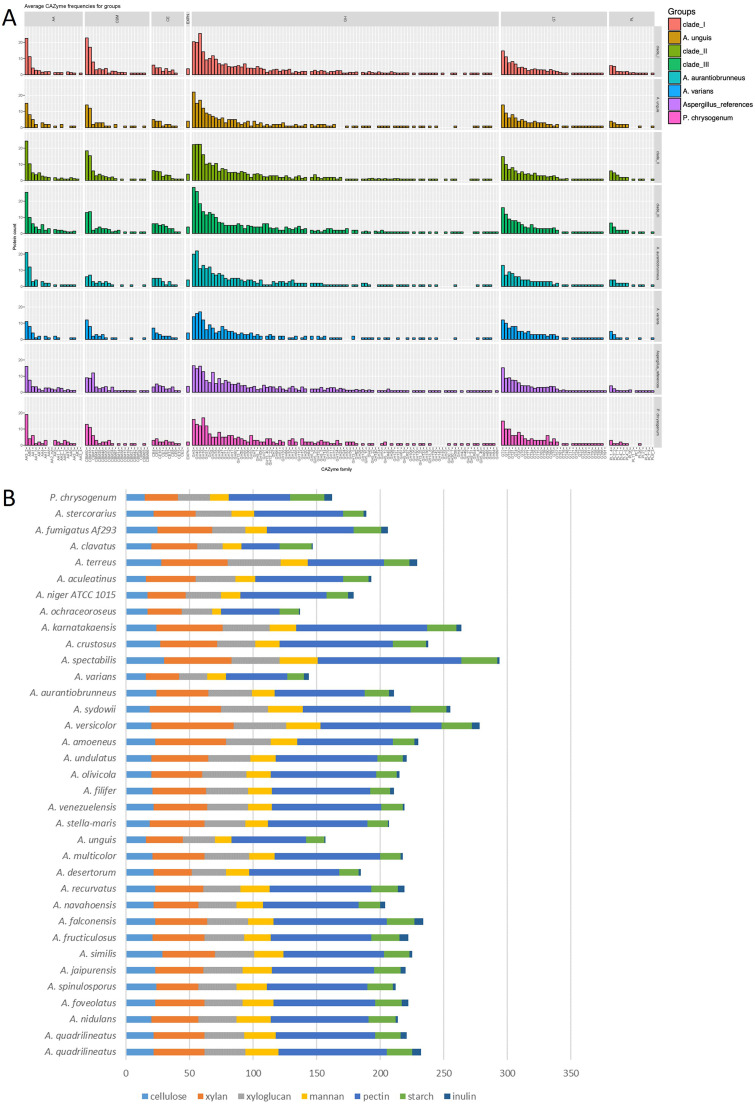
Comparative analysis of CAZymes in section *Nidulantes* and reference species. **A.** Bars show the average number of proteins in each clade. Top panels show CAZy families: auxiliary activities (AA), carbohydrate-binding molecules (CBM), carbohydrate esterases (CE), distant relatives of plant expansins (EXPN), glycoside hydrolases (GH), glycosyltransferases (GT), polysaccharide lyases (PL). **B.** Comparison of the number of genes related to degradation of different plant polysaccharides.

Overall, the species from section *Nidulantes* have a highly conserved set of plant biomass-targeting CAZyme genes (Fig. 5A). *A. versicolor* stands out with a higher number of genes than the other species, particularly due to a higher number of glycoside hydrolases (Fig. 5B, Table S1). In contrast, *A. unguis* and *A. varians* have a smaller gene set than the other species. The total number of CAZyme genes in section *Nidulantes* is in the same range as those of *A. terreus* and *A. niger*. However, the variation in this section is smaller than has been shown previously for section *Nigri* (Vesth et al., 2018) and section *Flavi* (Kjærbølling et al., 2020). In concordance with this, most species from section *Nidulantes* have highly similar growth profiles and they grow in general well on most tested substrates. Some exceptions to this are *A. falconensis, A. filifer, A. olivicola*, and *A. varians* that have a narrower set of substrates that supports good growth.

The highest diversity in growth between the species was found on the monosaccharides D-galactose, D-ribose, L-rhamnose, and D-galacturonic acid, and the polysaccharide inulin (Fig. S4). At this point, it is not clear what causes the variation in growth on these monosaccharides. It has been shown that many fungal ascomycetes are unable to germinate when D-galactose is present as the only carbon source (www.fung-growth.org) and it has been demonstrated for *A. niger* that this is due to the absence of D-galactose transport during germination (Fekete et al., 2004). The other three monosaccharides are so-called non-preferred carbon sources for most fungi, which could explain the higher variability with respect to growth between the species.

The growth difference on inulin is worth evaluating further as the presence of endoinulinase genes in their genome may be highly beneficial for fungi to grow on inulin (R.P. de Vries, unpublished data). The species that grow well on inulin are *A. quadrilineatus, A. foveolatus, A. similis, A. recurvatus, A. desertorum, A. crustosus, A. kanatakaensis, A. versicolor,* and *A. niger*, but interestingly some of these (*A. similis, A. desertorum*) do not appear to have an endoinulinase gene (Table S1). These genes were also not detected in the raw sequence data, which could suggest that these species developed an alternative approach to inulin degradation. Similarly, some species that contain an endoinulinase gene (*A. jaipurensis, A. fructiculosus, A. falconensis*) do not grow well on inulin, suggesting poor expression of the gene with inulin as a sole carbon source. Indications that the growth ability can be related to either expression of the genes or their presence or absence can also be found for other polysaccharides. Growth on xylan is very good for *A. unguis*, which has one of the lowest numbers of genes encoding putative xylan-acting enzymes, while *A. versicolor* with the highest number of these genes, does not show more than average growth on xylan. In contrast, poor growth of *A. unguis* on mannan correlates with the lowest number of putative mannan-acting genes in its genome, while the two species with highest number of these genes, *A. versicolor* and *A. sydowii*, both grow very well on this substrate. These results demonstrate that genomic comparison of fungi alone does not provide conclusive evidence that would aid in selection of species that produce efficient enzyme mixtures for degradation of specific polysaccharides. It may also indicate limitations in our current knowledge of plant cell walls across plant species and the comparatively small number of substrates available to perform growth experiments.

More detailed analysis of the absence and presence of putative genes encoding specific activities demonstrates diversity within section *Nidulantes*, as well as differences with other *Aspergillus* sections (Table S1). Examples of these are the absence of GH29 (xyloglucan) and CE15 (xylan) genes in most species of section *Nidulantes*, and variable numbers of GH26 (mannan), GH115 (xylan) and GH134 (mannan) genes within the section.

Some post-genomic studies showed that the diversity of plant biomass degradation between aspergilli extends beyond the genomic variation. When cultivated on the same plant biomass, eight aspergilli, including *A. nidulans*, produced highly diverse enzyme mixtures (Benoit et al., 2015). This could only be partially attributed to genomic differences, such as absence of genes in some species, as also proteins encoded by genes that were conserved among the species had different production profiles among these species. A later study set compared 18 species, now including three members of section *Nidulantes* (*A. nidulans, A. versicolor, A. sydowii*) (de Vries et al., 2017) and revealed differences in gene numbers per CAZy family between these three species, as well as highly diverse secretomes, when grown on plant biomass substrates. A more detailed analysis of the secretomes further dissected these differences, suggesting preference for different plant biomass polysaccharides of the three *Nidulantes* species (Mäkelä et al., 2018).

In conclusion, the genomic ability of *Aspergillus* section *Nidulantes*, is largely conserved and less diverse than observed for sections *Nigri, Flavi,* and *Usti* (Vesth et al., 2018; Kjærbølling et al., 2020). However, differences in the growth profiles of the species on plant biomass-related substrates and a large variety in the secretome of three species from the section when grown on plant biomass were observed. This suggests that the diversity in plant biomass degrading ability in the section *Nidulantes* is mainly at the post-genomic level.

### 3.10 Secondary metabolism shows high genetic diversity in section *Nidulantes* and suggests exchange between species

Aspergilli show a vast diversity of secondary metabolites (SMs), with rearrangements in secondary metabolite gene clusters (SMGCs) throughout species leading to new compounds. Hence, it was interesting to predict the presence of SMGCs and aggregate these into homologous families to describe the variety of SMGCs through species in section *Nidulantes*.

**Fig. 6:**
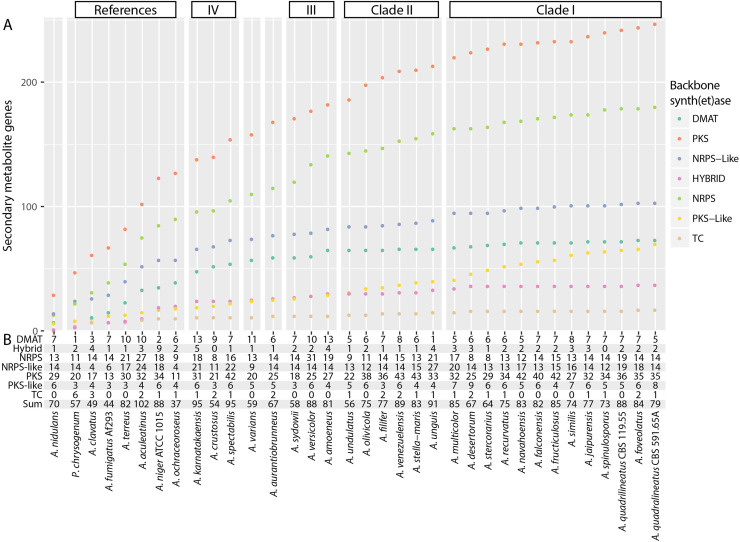
Overview of SMGC abundance and variation in section *Nidulantes* and reference species. A) Pan-genome of SMGCs. Species were added in order from left to right, and the number of new unique synthases and synthetases were added to the graph. B) Reference table of number of predicted synthases and synthetases. PKS: Polyketide synthase. NRPS: Non-ribosomal peptide synthetase. TC: Terpene cyclase. DMATS: Prenyltransferases.

Prediction of SMGCs identified 2154 synthetases across section *Nidulantes* (Fig. 6B). Taking *A. nidulans* SMGC families as the starting point, we performed a SM pan-genome analysis (Fig. 6A) to evaluate the genetic variation across the section.

Examining the pan-genome (Fig. 6A), we see that the amount of unique SMGCs found within section *Nidulantes* is for PKSs, PKS-likes, and NRPSs similar to the combined diversity of the included diverse reference species. For all three types, the number of unique synth(et)ase cluster roughly doubles compared to the reference species (Fig. 6A). We see this as an indication of potential horizontal gene transfer, and as section *Nidulantes* having a diverse set of NRP and PKs.

**Fig. 7:**
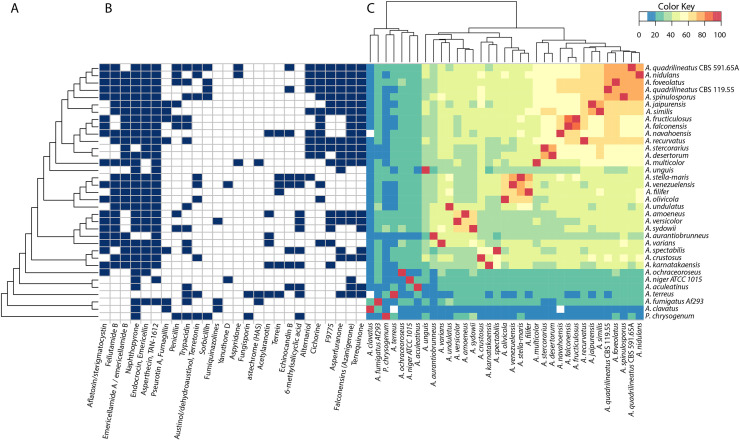
SMGC diversity throughout the dataset. Left: Annotation of SMGC families with known compounds from the MIBiG database. Right: Comparison of SMGC families between species. The heatmap shows the amount of shared SMGC families in percent indicated by cell color. Column dendrogram of organisms is clustered hierarchically according to SMGC families.

To further evaluate genetic diversity of secondary metabolism, we sorted all SMGC into families of clusters across the genomes, showing that 603 families were representative of the set, and 71 % (433) of these are unique to a single species (Fig. S5). We also see that the species in section Nidulantes share 40–50 % of SMGCs with other species, and the patterns of shared clusters is largely in accordance with phylogeny (Fig. 7). This strongly suggests the shared gene clusters to be early acquisitions, while the SMGCs found in single species are late acquisitions, and is thus in support of the theories of highly active HGT of SMGC in aspergilli.

By clustering the species by their shared SMGC content, we can identify subgroups inside the section (Fig. 7) which are to a large extent in support of the phylogenetic clades I-V (Fig. 1). We also see species to have at least 20–30 % similarity, which is more than in section Nigri, where species have as little as 10–20 % similarity (Vesth et al., 2018). This supports that clade III and IV are inside section *Nidulantes* which has been discussed (Hubka et al., 2016; Kocsubé et al., 2016), and the concept of *Nidulantes* as a phylogenetically distinct section.

However, it is also distinctly clear that the SMGC profiles of *A. fructiculosus* and *A. falconensis* are high similar (90–100 %) strongly suggesting that they should be one species.

### 3.11 Genetic dereplication links gene clusters to secondary metabolites and shows indications of recent gene transfer from *A. fumigatus*

Analytical studies of aspergilli have shown a large chemical diversity through sections (Frisvad and Larsen, 2015) with only a small fraction of compounds being conserved over large phylogenetic distances. In many cases SMs have been used to identify species (Frisvad et al., 2008). Hence, we were interested in linking genes to metabolites.

We developed a genetic dereplication method which used a conservative guilt-by-association approach to connect known clusters from the MIBiG database to the SMGC families.

Our analysis revealed several conserved clusters. The sterigmatocystin, fellutamide B (Yeh et al., 2016), emericellamide, YWA, endocrocin (Lim et al., 2012), emericellin as well as the asperthecin SMGC (Szewczyk et al., 2008) have related gene clusters in almost all species of section *Nidulantes*. Thus, they are characteristic for the section. The alternariol, cichorine, F9775 (orsellinic acid), asperfuranone, azanigerone and terrequinone cluster homologs are present in many members of clade I and scattered over clades III and IV. This pattern indicates that the gene clusters have been acquired before differentiation of the *Nidulantes* section, then lost in some species. This would fit with the similarities of the falconensins and azanigerones, which were described in section *Nigri* (Zabala et al., 2012).

We further identified SMGCs that suggest recent acquisition by horizontal gene transfer. A gene cluster in *A. multicolor* shows high synteny conservation to the hexadehydro-astechrome (HAS) gene cluster of *A. fumigatus*, whose product increases virulence (Yin et al., 2013), and to the astechrome gene cluster in *A. terreus* (Fig. 7). The latter cluster only produces astechrome since it misses a flavin adenine dinucleotide (FAD) binding enzyme (Yin et al., 2013; Bok et al., 2015). The *A. multicolor* cluster, however, contains the FAD gene, suggesting it produces HAS. To confirm the hypothesis of HGT, we identified a syntenic site to the area surrounding the HAS homolog-*locus* of *A. multicolor* in *A. nidulans* (Fig. S6). We sustained our hypothesis of HGT comparing a ML phylogeny of HAS homologs against homologs of the conserved NRPS, SidC. Homologs of SidC in *A. multicolor, A. fumigatus* and *A. terreus* show a greater distance than their HAS homologs (Fig. S6). Furthermore, the distances shown for their HAS homologs resemble that of conserved NRPSs inside the same section, suggesting recent acquisition of the HAS cluster. Thus, we show evidence for HGT of the HAS NRPS from *A. fumigatus* to *A. multicolor*.

## 4 Conclusions

We *de novo* sequenced species in section *Nidulantes* and related their genetic content to the genome of the model organism *A. nidulans*. Our analysis shows that general regulators characterized in *A. nidulans* are distributed throughout its section. Protein family analysis indicated that in some cases we identified paralogs, pointing to a different function. In the case of LaeA paralogs this could mean that they function on different SMGCs than LaeA. Key cell cycle, cytoskeletal, polarity, and sexual development genes are present throughout all species confirming *A. nidulans* role as model organism, as already assumed since the early studies of Pontecorvo et al. (1953). Protein families provide insights into clade specific adaptations with loss of proteins in clade III and IV. Gene cluster predictions were confirmed by data on analytical studies in the case of sterigmatocystin and karnatakafurans.

Our genetic dereplication analysis showed that elucidated gene clusters of *A. nidulans* are biased towards section *Nidulantes* and clade I. Despite this, it is still informative to mine closely related species for new SMGC. As the analysis of non-redundant SMGC added per genome shows, we can expect that the species sequenced in this study will supply novel SMGCs. Although most new SMGCs are being discovered from aspergilli from different sections, closely related species can still yield a vast amount of non-redundant PKSs and NRPSs.

## Funding sources

The work (proposal 10.46936/10.25585/60001025) performed at the US Department of Energy (DOE) Joint BioEnergy Institute and the US DOE Joint Genome Institute (https://ror.org/04xm1d337), a DOE Office of Science User Facility, is supported by the Office of Science of the U.S. Department of Energy operated under Contract No. DE-AC02-05CH11231 between Lawrence Berkeley National Laboratory and the US DOE. MRM acknowledges funding from Research Council of Finland (grant no. 314102) and Novo Nordisk Foundation (grant no. NNF21OC0067087). MRA, IK, JLN, ST and TCV gratefully acknowledge funding from the Villum Foundation, Grant VKR023437. MRA, IK and TCV acknowledge funding from the Danish National Research Foundation, grant number DNRF137. MRA acknowledges support from the Jorck Foundation. PSD thanks the Wellcome Trust (UK) for funding (no. 219551/Z/19/Z).

## CRediT authorship contribution statement

**Sebastian Theobald:** Conceptualization, Methodology, Formal analysis, Investigation, Writing – original draft. **Tammi Vesth:** Conceptualization, Methodology, Formal analysis, Writing – original draft. **Jane L. Nybo:** Methodology, Formal analysis. **Jens C. Frisvad:** Formal analysis, Writing – original draft, Writing – review & editing. **Inge Kjærbølling:** Methodology. **Stephen Mondo:** Formal analysis. **Kurt LaButti:** Formal analysis. **Sajeet Haridas:** Formal analysis. **Robert Riley:** Formal analysis. **Alan A. Kuo:** Formal analysis. **Asaf A. Salamov:** Formal analysis. **Jasmyn Pangilinan:** Formal analysis. **Anna Lipzen:** Formal analysis. **Maxim Koriabine:** Formal analysis. **Mi Yan:** Formal analysis. **Kerrie Barry:** Formal analysis. **Alicia Clum:** Formal analysis. **Ellen K. Lyhne:** Investigation. **Elodie Drula:** Formal analysis. **Ad Wiebenga:** Investigation. **Astrid Müller:** Writing – original draft. **Ronnie J.M. Lubbers:** Investigation. **Roland S. Kun:** Investigation. **Ana Carolina dos Santos Gomes:** Investigation. **Miia R. Mäkelä:** Formal analysis, Writing – original draft, Writing – review & editing. **Bernard Henrissat:** Formal analysis, Writing – review & editing. **Blake A. Simmons:** Methodology. **Jon K. Magnuson:** Methodology, Formal analysis. **Jakob B. Hoof:** Formal analysis. **Uffe H. Mortensen:** Methodology. **Paul S. Dyer:** Conceptualization, Formal analysis, Writing – original draft, Writing – review & editing. **Michelle Momany:** Conceptualization, Formal analysis, Writing – original draft, Writing – review & editing. **Thomas O. Larsen:** Formal analysis, Writing – review & editing. **Igor V Grigoriev:** Methodology, Formal analysis, Writing – original draft, Writing – review & editing. **Scott E. Baker:** Conceptualization, Writing – original draft. **Ronald P. de Vries:** Conceptualization, Formal analysis, Writing – original draft, Writing – review & editing. **Mikael R. Andersen:** Conceptualization, Writing – original draft, Writing – review & editing.

## Declaration of competing interest

The authors declare the following financial interests/personal relationships which may be considered as potential competing interests:

Li Xu reports financial support was provided by Chinese Scholarship Council. If there are other authors, they declare that they have no known competing financial interests or personal relationships that could have appeared to influence the work reported in this paper.

## Data Availability

all data is publicly available through mycocosm.

## Appendix Supplementary materials

Image, application 1

Image, application 2

Image, application 3

Image, application 4

Image, application 5

Image, application 6

Image, application 7
