# Supplementary figures and images for: Comparative genomics of Aspergillus nidulans and section Nidulantes
Source: Curr Res Microb Sci. 2025 Jan 16;8:100342. doi: 10.1016/j.crmicr.2025.100342 (PMC11787670; doi:10.1016/j.crmicr.2025.100342)

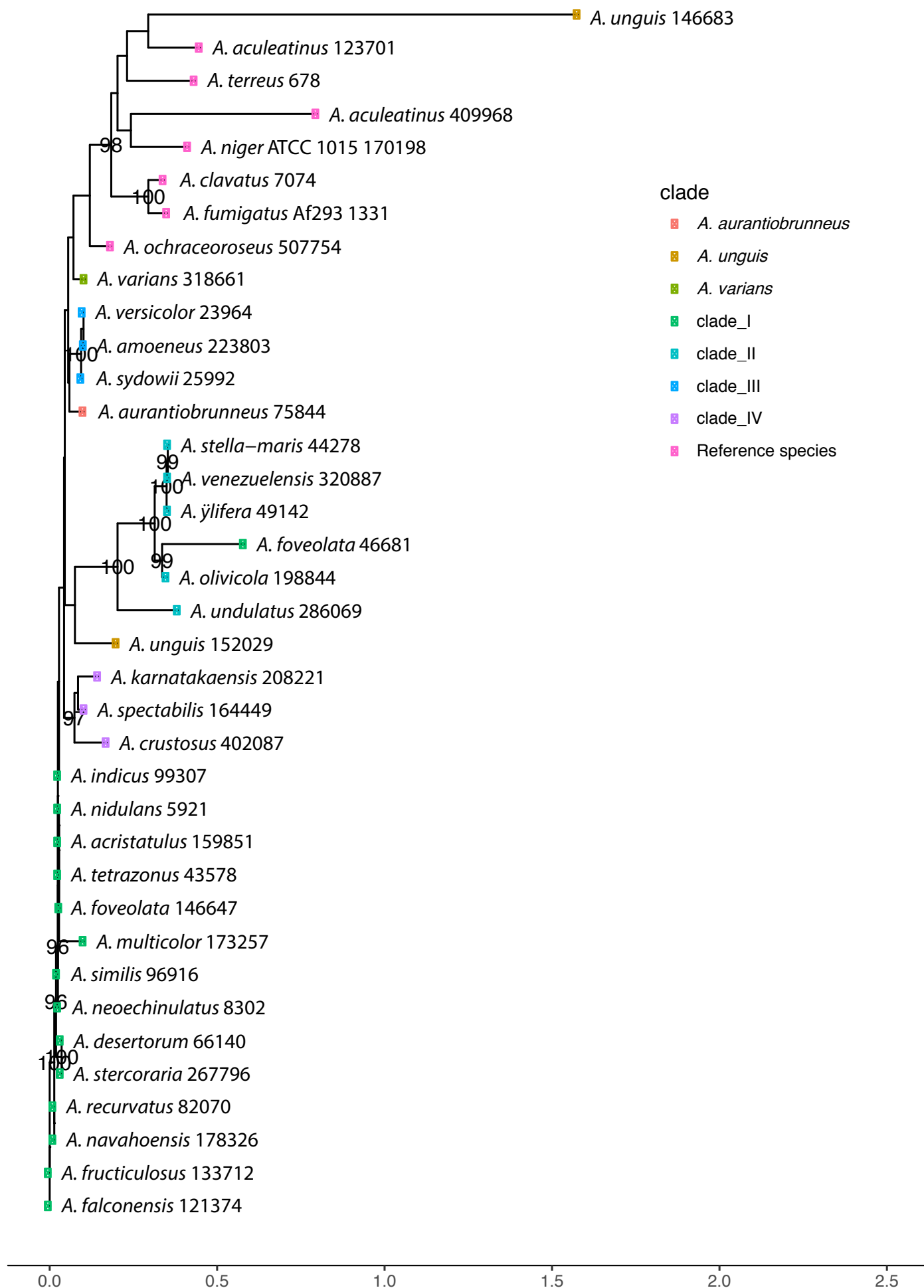

**Figure S1.** Phylogenetic tree of LaeA from all tested species.

Supplement: Supplementary file 1 [file mmc1.pdf]

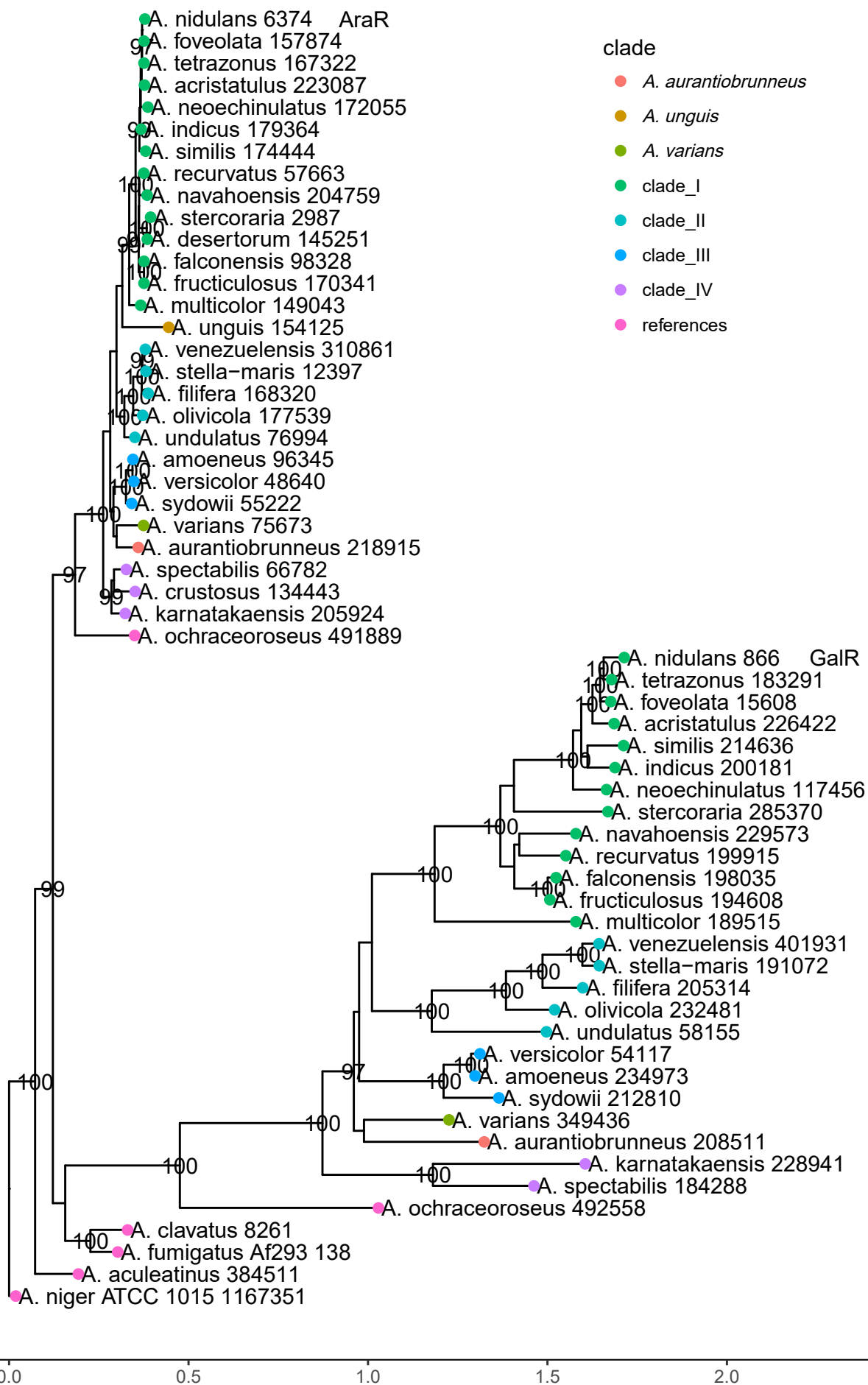

**Figure S2.** Phylogenetic tree of AraR and GalR from all tested species.

Supplement: Supplementary file 2 [file mmc2.pdf]

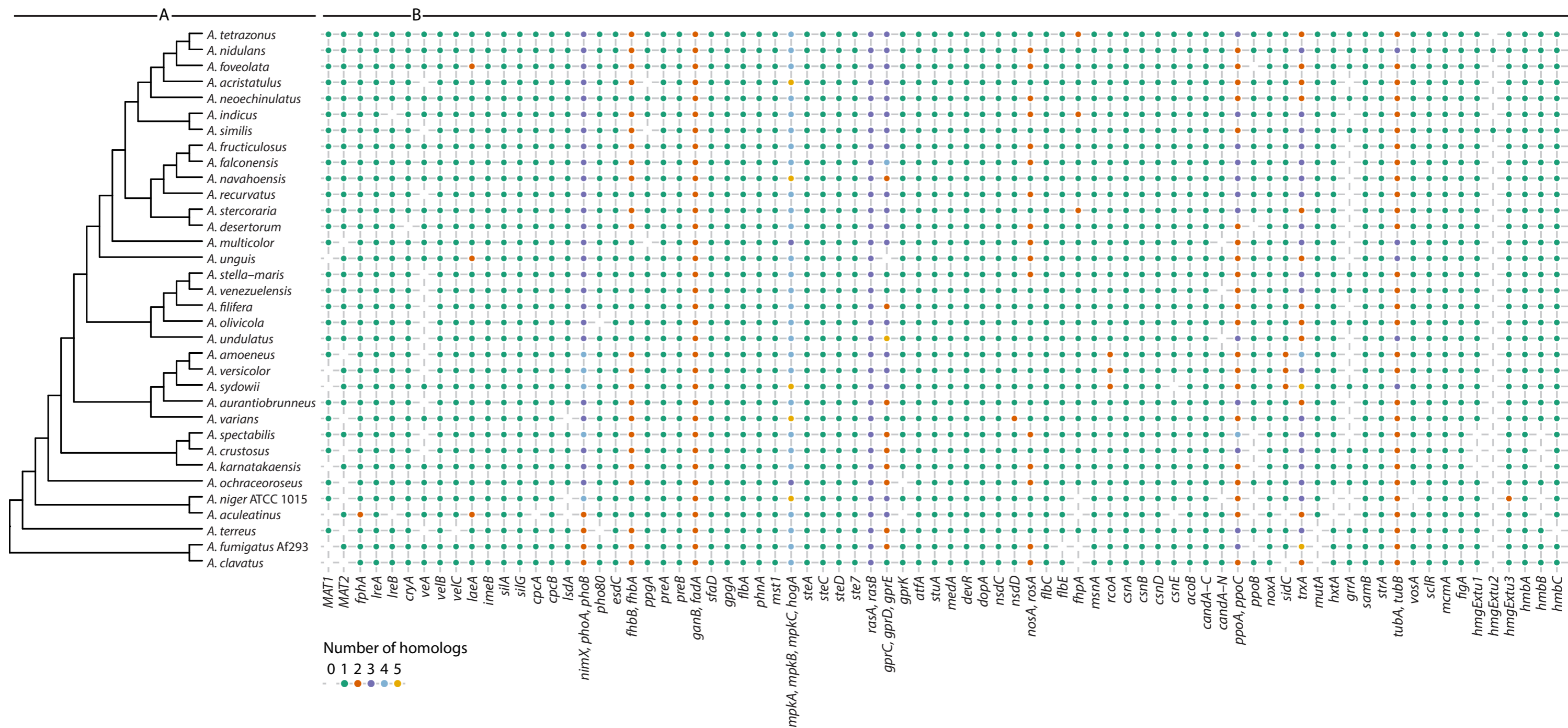

**Figure S3.** Overview of the presence of genes related to sexual development in all tested species.

Supplement: Supplementary file 3 [file mmc3.pdf]

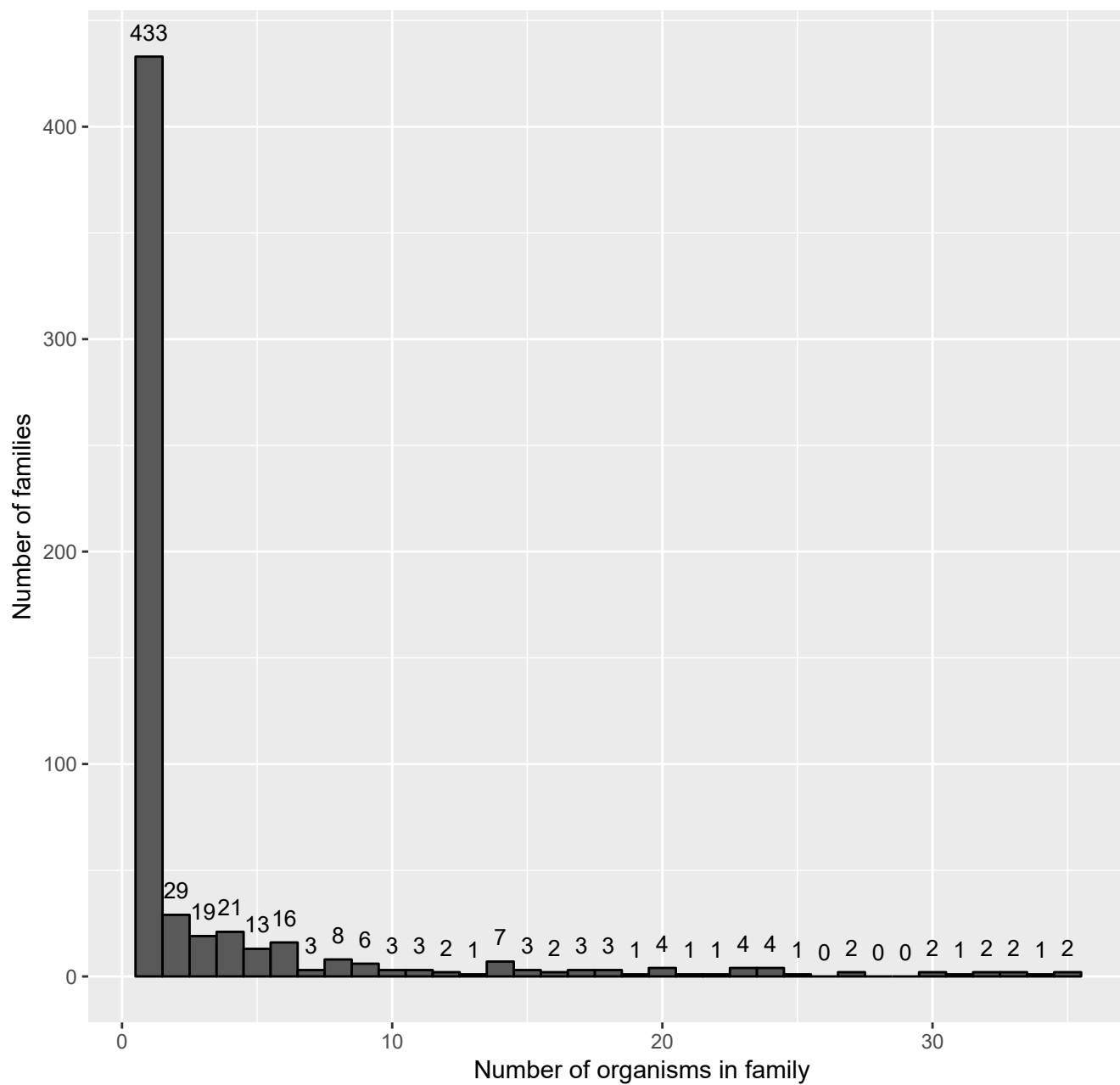

**Figure S5.** Number of SMGC families with clusters in x number of organisms.

Supplement: Supplementary file 5 [file mmc5.pdf]
