# Supplementary material for: Comparative genomics of Aspergillus nidulans and section Nidulantes
Source: Curr Res Microb Sci. 2025 Jan 16;8:100342. doi: 10.1016/j.crmicr.2025.100342 (PMC11787670; doi:10.1016/j.crmicr.2025.100342)
Supplement: Supplementary file 4 [file mmc4.pdf]

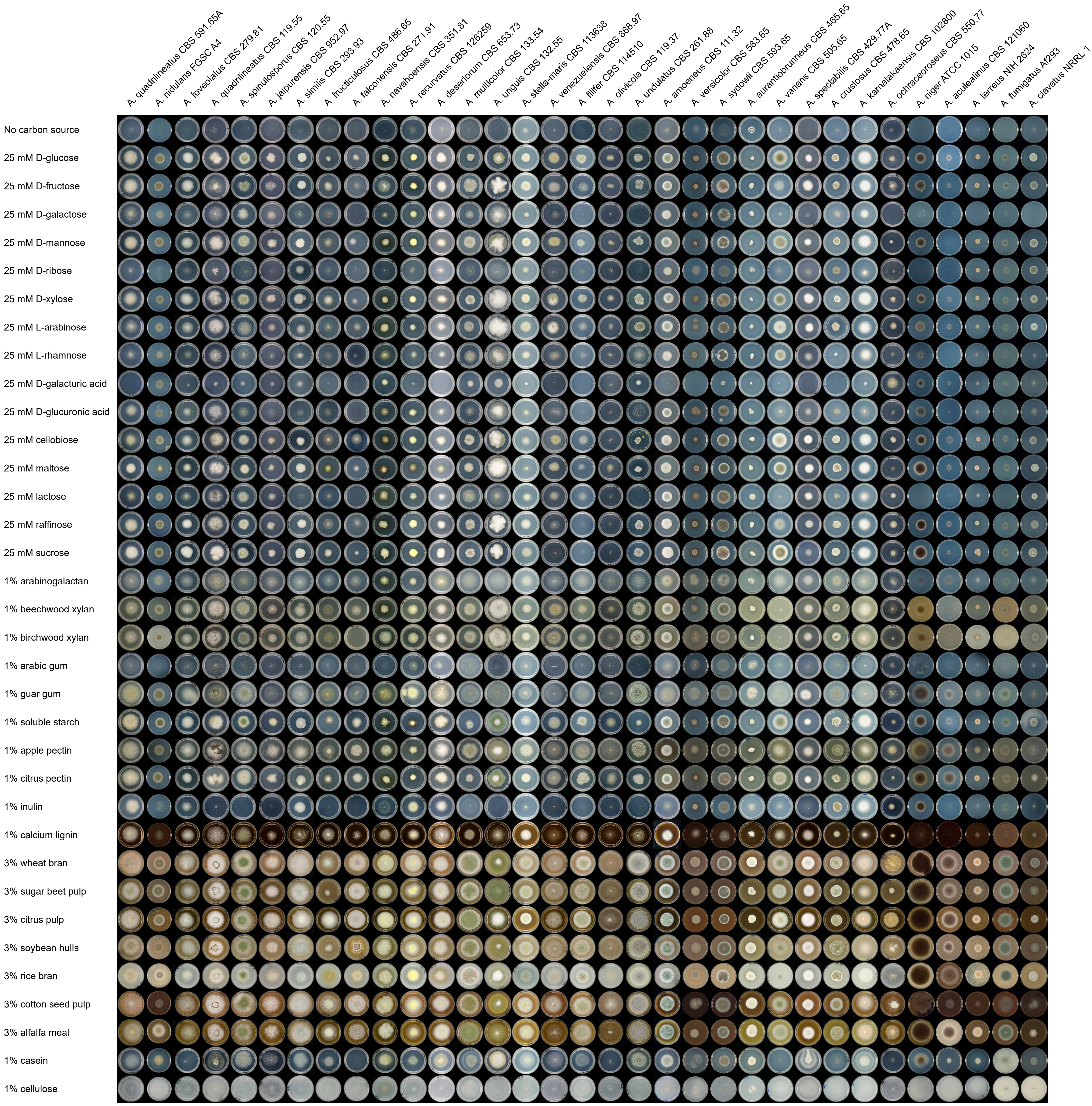

Figure S4. Growth profiles of all selected species on monosaccharides (25 mM), oligosaccharides (25 mM), polysaccharides (1% w/v) and crude plant biomass substrates (1% w/v). Growth was analyzed after 5 days incubation at 30 degrees Celcius.
