# Supplementary material for: Comparative genomics of Aspergillus nidulans and section Nidulantes
Source: Curr Res Microb Sci. 2025 Jan 16;8:100342. doi: 10.1016/j.crmicr.2025.100342 (PMC11787670; doi:10.1016/j.crmicr.2025.100342)
Supplement: Supplementary file 6 [file mmc6.pdf]

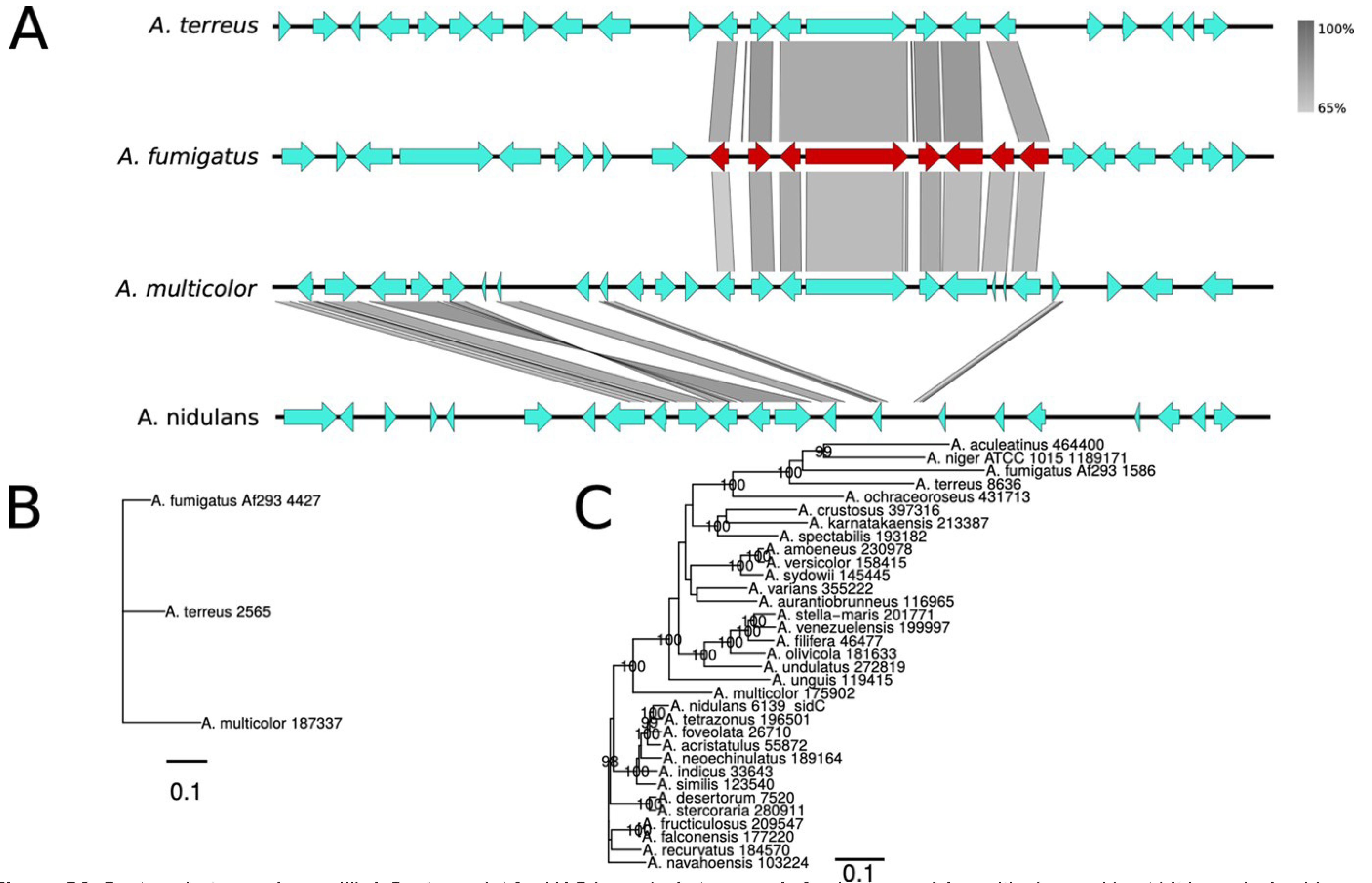

Figure S6. Synteny between Aspergilli. A Synteny plot for HAS locus in A. terreus, A. fumigatus and A. multicolor, and best hit locus in A. nidulans. hasA-H (55) are conserved in A. multicolor, while hasG is missing in A. terreus. Thus, we expect A. multicolor to synthesize full HAS (A. terreus only produces astechrome according to Bok et al. (56)). A. nidulans contains a region which is syntenic to the upstream region of the HAS locus. Hence, we expect that the HGT event occurred at Chr VII of A. nidulans. Homologs for these HAS proteins were only found in the family. B ML phylogeny of HAS and (C) sidC-homolog NRPSs. Protein sequences were aligned using Clustal Omega and trimmed using trimal prior to Maximum Likelihood analysis using iqtree. HAS homologs (left) show phylogenetic proximity.
